# Management of Pediatric Pulmonary Vein Stenosis

**DOI:** 10.1016/j.jscai.2022.100391

**Published:** 2022-06-30

**Authors:** Ryan Callahan, Brian H. Morray, Russel Hirsch, Christopher J. Petit

**Affiliations:** a Department of Cardiology, Boston Children’s Hospital and Harvard Medical School, Boston, Massachusetts; b Division of Pediatric Cardiology, Seattle Children’s Hospital and University of Washington School of Medicine, Seattle, Washington; c Heart Institute, Cincinnati Children’s Hospital and University of Cincinnati College of Medicine, Cincinnati, Ohio; d Division of Pediatric Cardiology, Morgan Stanley Children’s Hospital, NewYork-Presbyterian Hospital and Columbia University Vagelos College of Physicians and Surgeons, New York, New York

**Keywords:** congenital heart disease, management, pulmonary vein stenosis, therapy

## Abstract

Pediatric intraluminal pulmonary vein stenosis has evolved into a chronic illness, with improving survival. Although significant knowledge gaps remain, medical providers have found success in the management of patients with pulmonary vein stenosis using a comprehensive multimodality treatment strategy. This review discusses the core principles employed by 4 centers dedicated to improving pulmonary vein stenosis outcomes, including how to make the diagnosis, educating the family, treatment strategy, the importance of surveillance, and the management of symptoms and comorbidities.

## Highlights

• Pediatric intraluminal pulmonary vein stenosis is a chronic illness
• The management of pulmonary vein stenosis requires a multimodality treatment strategy
• Dedicated programs use a team-based approach, including multiple subspecialists
• Regular surveillance testing is critical for vein preservation
• Comorbidities are common and can affect pulmonary vein stenosis therapy and overall prognosis

## Central Illustration

**Figure:**
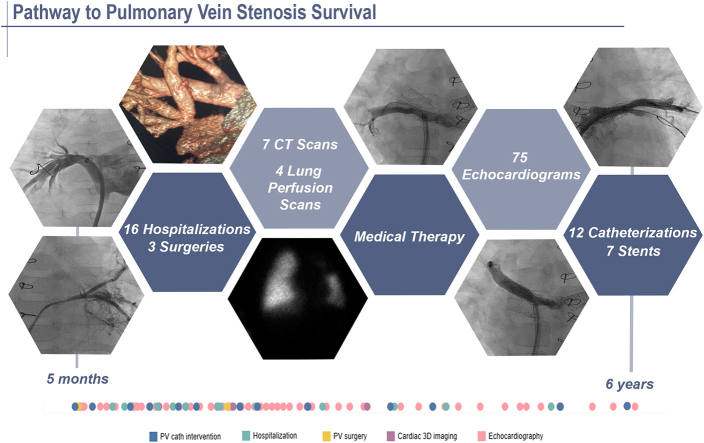

Initially labeled a fatal disease with no worthy treatment options, pediatric intraluminal pulmonary vein stenosis (PVS) has evolved into a chronic illness, with improving survival. Although there is no established treatment algorithm that can be successfully applied to all patients, institutions dedicated to optimizing PVS outcomes have jointly recognized the core principles necessary for the treatment of patients with PVS. This article aims to present the shared real-world strategies from 4 institutions in the United States in the management of PVS. For a comprehensive literature review of PVS, please refer to the several recently published reviews.^1^, ^2^, ^3^, ^4^, ^5^, ^6^

## Overview

Pediatric intraluminal PVS is a disease of pulmonary vein wall thickening. Specifically, hyperplasia (neointimal proliferation) of myofibroblast-like cells in a loose myxocollagenous matrix, which typically originates at the venoatrial junction, can extend distally or upstream into the intrapulmonary veins and lead to lumen obliteration.^7^, ^8^, ^9^ This fibromyxoid proliferation causing luminal narrowing is commonly seen at the location of vein distortion by the surrounding anatomy, leading some investigators to hypothesize that disturbed flow and elevated wall shear stress (WSS) may play a role in the development of PVS.^10^, ^11^, ^12^, ^13^ Although the exact incidence of patients with PVS is unknown, there is a perception of increasing numbers potentially due to an increase in awareness, improved survival of premature infants, and evolving definitive and palliative surgeries for complex congenital heart disease. The incidence of ex-full-term patients with structurally normal hearts presenting in the first few months of life with PVS appears to be stable.

## Presentation

Pediatric PVS typically presents in the initial years of life, most commonly in infancy. The clinical sequelae of PVS are the direct result of pulmonary venous hypertension from narrowing of the pulmonary veins. When the high pulmonary venous pressure exceeds plasma oncotic pressure, then transudation of protein-poor fluid crosses the pulmonary endothelium into the pulmonary interstitium and alveoli. If the lymphatic system fails to clear the fluid, then pulmonary edema occurs, resulting in ventilation-perfusion mismatch, hypoxemia, tachypnea, and work of breathing. Pulmonary venous hypertension can be combined with secondary postcapillary pulmonary hypertension (PH), both of which increase right ventricular (RV) afterload causing low cardiac output and compensatory tachycardia. The resultant excessive cardiopulmonary caloric expenditure paired with symptoms of right-sided heart failure (feeding intolerance, agitation, etc.) leads to failure to thrive. Older children and young adults typically present with exercise intolerance as a result of RV hypertension and have a history of a single or multiple episodes of “pneumonia.” Rarely, patients can present with hemoptysis/hematemesis secondary to pulmonary vein atresia and the formation of bronchial or esophageal varices from pulmonary venous hypertension and venovenous collateral formation.^14^, ^15^, ^16^ In severe cases, usually provoked by an additional insult such as a lower respiratory illness or transcatheter intervention, hemoptysis from alveolar hemorrhage can occur.^17^

**Figure 1:**
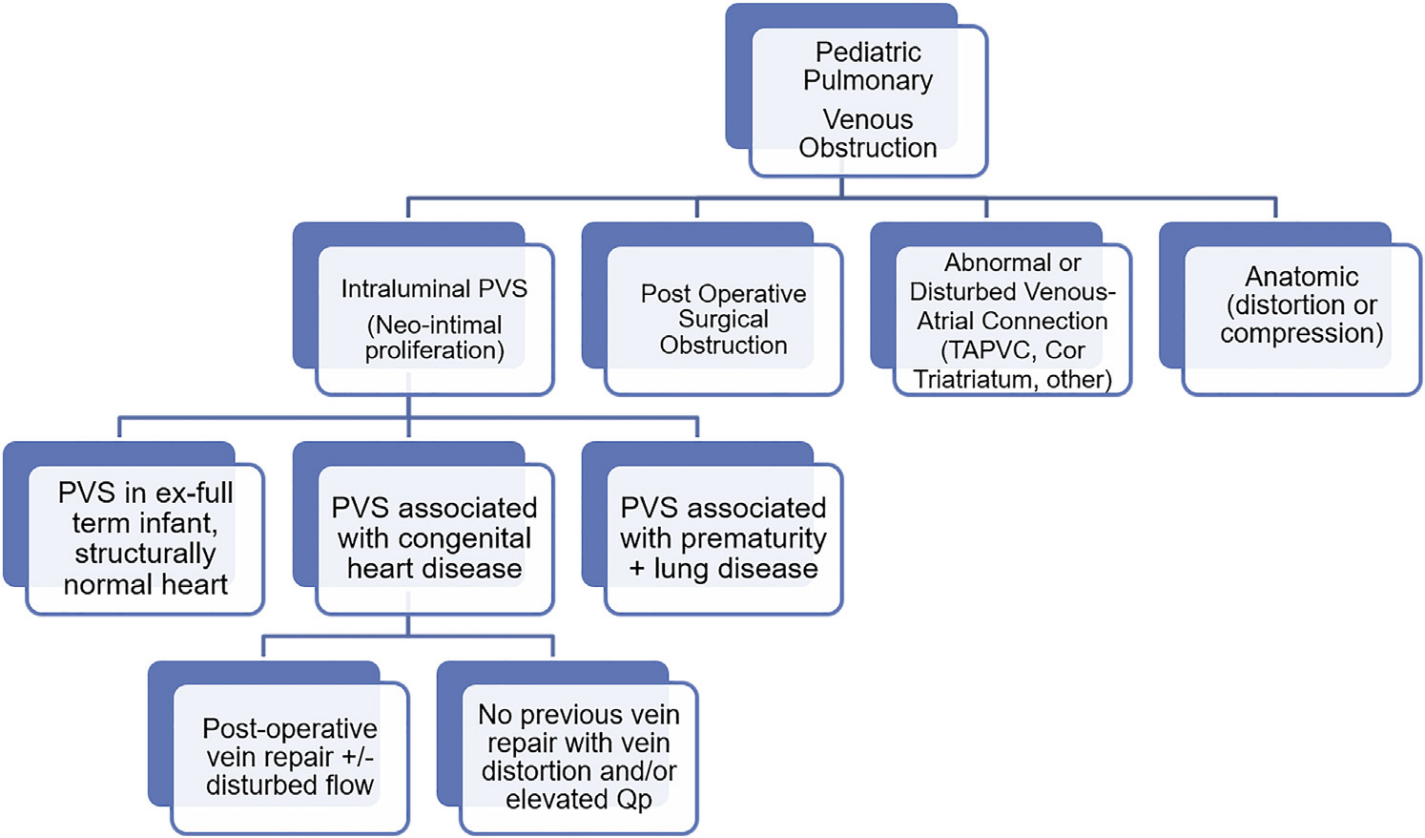
**Tree diagram of pediatric pulmonary venous obstructions**. The tree diagram demonstrates the mechanisms of pulmonary venous obstruction, including intraluminal PVS. The various types of PVS are outlined. PVS, pulmonary vein stenosis; Qp, pulmonary blood flow; TAPVC, total anomalous pulmonary venous connection.

There are several populations of patients who are at risk of PVS development (Figure 1). The ex-full-term infant with a structurally normal heart presenting within the first few months of life with failure to thrive and respiratory failure is an aggressive subtype and can occur in siblings, although, to this point, PVS has not been linked to a causal gene. Premature infants, typically with a left-to-right shunt (ie, patent arterial duct, atrial septal defect, ventricular septal defect), earlier gestation, bronchopulmonary dysplasia (BPD), and/or necrotizing enterocolitis, are at risk of PVS.^18^, ^19^, ^20^ Earlier gestation and severe PH are associated with a higher risk of progressive multivessel PVS.^21^ The location of PVS within the pulmonary veins in this population is not necessarily at the venoatrial junction but rather at the area of pulmonary vein distortion from the surrounding anatomy—specifically, the left upper pulmonary vein from the left posterior bronchus, the left lower pulmonary vein between the heart mass and aorta, and the right upper pulmonary vein from the right pulmonary artery, with the usual sparing of the right lower pulmonary vein. The pericardial reflection may also play a role in fixing the pulmonary veins, allowing for further vein distortion. The distortion progresses secondarily as thoracic conditions worsen: right pulmonary artery dilation in worsening PH, left bronchial dilation in chronic ventilation, and heart rotation stretching left lower pulmonary vein over aorta in chronic dependent atelectasis.^10^^,^^12^ “Secondary” or “postoperative” PVS is a well-known phenomenon occurring in patients born with anomalous pulmonary venous connections or obstructive atrial tissue (ie, cor triatriatum) requiring surgical repair.^22^ Delineating the mechanism of postoperative pulmonary venous obstruction is critical, as the neointimal proliferation pathognomonic for PVS within the individual vessels does not always occur. Rather, the mechanism of obstruction includes an inadequate surgical confluence anastomosis, surgical scar, pulmonary vein distortion, and/or obstructive residual atrial tissue. Patients with congenital heart disease and normally connected pulmonary veins remain the most heterogeneous population at risk of PVS development. Trends can be seen in subgroups, such as an elevated overall or lobar Qp (pulmonary blood flow) as a result of a left-to-right shunt or contralateral pulmonary artery stenosis, respectively, flowing through a vulnerable vein (ie, common pulmonary vein). There is a growing concern over the incidence of PVS occurring following orthotopic heart transplantation and is postulated to be the result of an increase in Qp (compared with that before operation) and the integrity of the left atrium donor/recipient anastomosis. Lastly, there may be patients with a genetic susceptibility to PVS development, either innately (ie, Smith-Lemli-Opitz syndrome) or as a result of other comorbidities associated with the disease (ie, trisomy 21’s association with a left-to-right shunts, hypotonia, aspiration, apnea).^23^, ^24^, ^25^, ^26^

## Diagnosis

### Chest radiography

**Figure 2:**
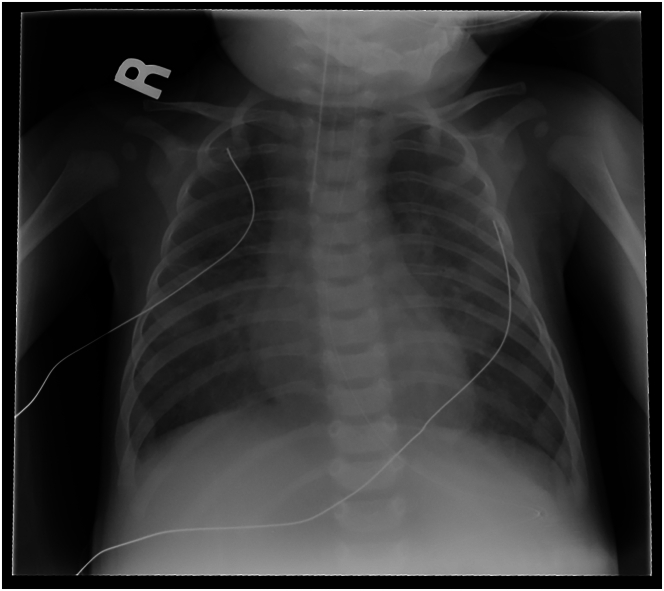
**Chest radiograph of a 3-month-old child presenting with respiratory failure secondary to multivessel pulmonary vein stenosis**. The image demonstrates a bilateral pattern of interstitial opacities as a result of pulmonary venous hypertension and interstitial edema. R, right.

Common chest radiography findings include interstitial and reticular opacities as a result of venous congestion and fluid in the alveolar space (Figure 2).^27^ There is typically asymmetric vascularity between lungs and abnormal distribution within each lung, with pulmonary edema more prominent in lung fields where vascularity is more prominent.^28^ The heterogeneous patterns of opacities and vascularity occur as a result of blood flow redistribution to unaffected or less affected veins. The chest radiography findings can be confounded by the sequelae of congenital heart disease and lung disease such as BPD.

### Echocardiography

Ultrasound evaluation of the pulmonary veins is typically the first comprehensive imaging modality that raises suspicion for PVS and can be employed as a longitudinal screening tool for initial diagnosis and for following disease progression. Mean vein gradients of 2 to 5 mm Hg, loss of phasic flow, and decreased pulsatility on spectral Doppler are all used as criteria for the diagnosis of PVS.^4^^,^^29^, ^30^, ^31^ However, echocardiography is limited in that it is unable to provide sufficient information on vein caliber, and the presence of distal disease and the spectral Doppler assessment of gradients may not accurately reflect the degree of obstruction if the blood flow to that segment of the lung is significantly reduced. Thus, it is typically used in conjunction with other imaging modalities, such as nuclear medicine lung perfusion (LP) scans and computed tomography angiography (CTA).

### Nuclear medicine LP scan

**Figure 3:**
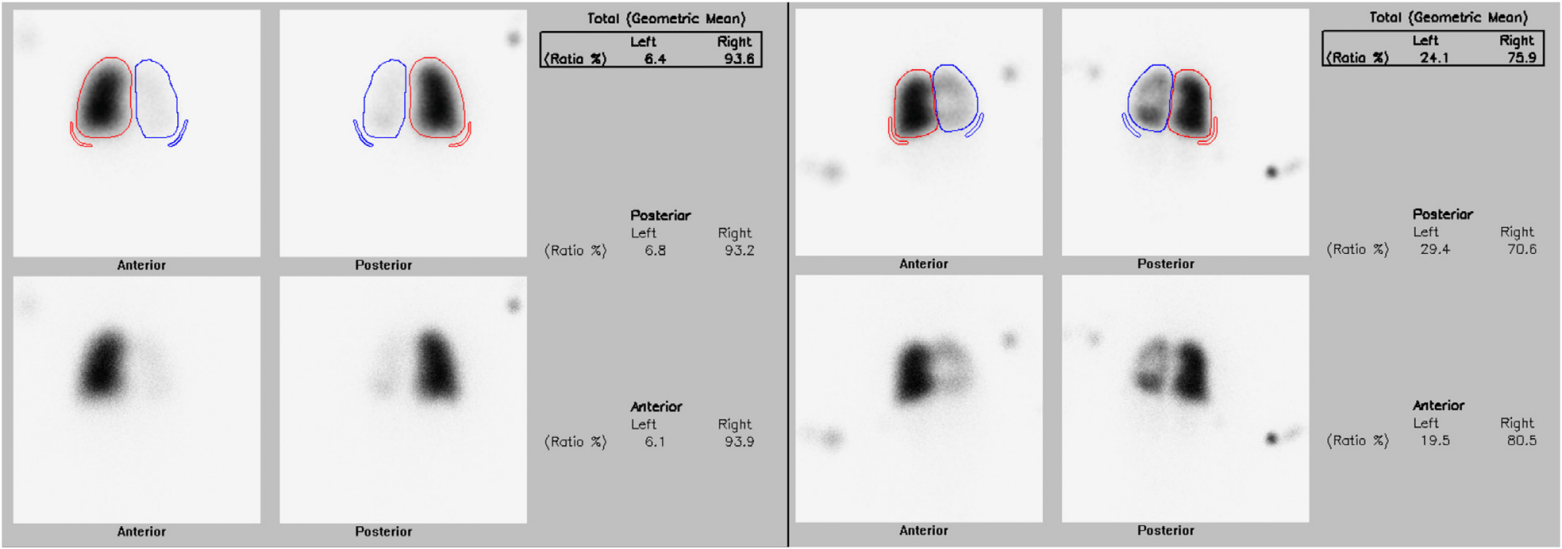
**Nuclear medicine lung perfusion scan obtained before and after transcatheter intervention**. The lung perfusion scan demonstrates decreased flow to the left lung secondary to severe left common pulmonary vein stenosis (left). There is improved flow to the left lung following transcatheter intervention (right).

Nuclear medicine LP scans are commonly used to diagnose PVS and monitor the progression of vein disease after treatment (Figure 3). LP findings correlate with angiographic findings in the diagnosis of PVS.^32^ The radiation dose associated with the study depends on age, ranges from 0.4 to 0.9 mSv, and is minimized only when the anterior/posterior projections are obtained. LP scans are frequently used in conjunction with echocardiography to determine whether the changes in gradient measured on echocardiography are related to either a decrease or increase in lobar perfusion. For example, a gradient of 3 mm Hg by echocardiography may be significant if the flow is redistributed to other lobes. Further, a gradient of 8 mm Hg by echocardiography may be insignificant if the lobe receives the majority of the cardiac output from contralateral vein stenosis/atresia and the Doppler pattern remains phasic.

### CTA

CTA is valuable in the initial diagnosis of PVS and for procedural planning and surveillance monitoring of disease progression. CTA provides excellent anatomic detail with a high spatial resolution that can determine the number of vessels involved, characterize the extent of disease in each vessel and in the case of previous stent implantation, determine the extent of in-stent restenosis. Radiation doses range from 0.5 to 3 mSv depending on the equipment and technique used. Unlike conventional angiography, CTA can characterize the relationship of important surrounding vascular and soft tissue structures with the pulmonary veins and identify potential sites of venous distortion. Patients with PVS have characteristic extravascular thoracic multidetector CTA findings, including ground-glass opacity, septal thickening, pleural thickening, and an ill-defined, mildly heterogeneously enhancing, noncalcified soft tissue mass surrounding the affected veins.^33^ Further, CTA allows for the application of a scoring system of PVS severity that has been demonstrated to correlate well with lung disease severity and with PVS-related mortality.^34^^,^^35^

### Cardiac magnetic resonance imaging

Cardiac magnetic resonance (CMR) imaging is less commonly employed as a screening or surveillance tool in the management of PVS. It is limited by the duration of the examination, the need for sedation, artifact from previous stent implantation as well as the limited spatial resolution it provides, particularly in smaller patients. Nevertheless, centers with expertise have adopted CMR imaging for PVS surveillance and characterization of PVS severity and disease location.^13^ CMR imaging does provide functional data (such as phase-encoded velocity mapping to calculate flow) that echocardiography or CTA do not. CMR imaging also allows for more quantitative assessments of RV function and provides estimates of differential pulmonary blood flow.^36^

### Invasive intraluminal imaging

Intravascular ultrasound (IVUS) can visualize vessel wall architecture, making it a potentially valuable tool in the evaluation and treatment of PVS. IVUS has been demonstrated to be safe in infants, can assist in determining the mechanism of obstruction, identify features associated with restenosis, and guide interventions.^37^^,^^38^ In addition, the histologic correlation of diseased veins with known PVS was performed using in vivo and in vitro samples, further characterizing the ultrasonographic features of PVS.^9^ However, the primary limitation of IVUS in pediatric catheterization laboratories is the cost associated with acquiring the necessary equipment and a lack of experience in interpreting the images.

**Figure 4:**
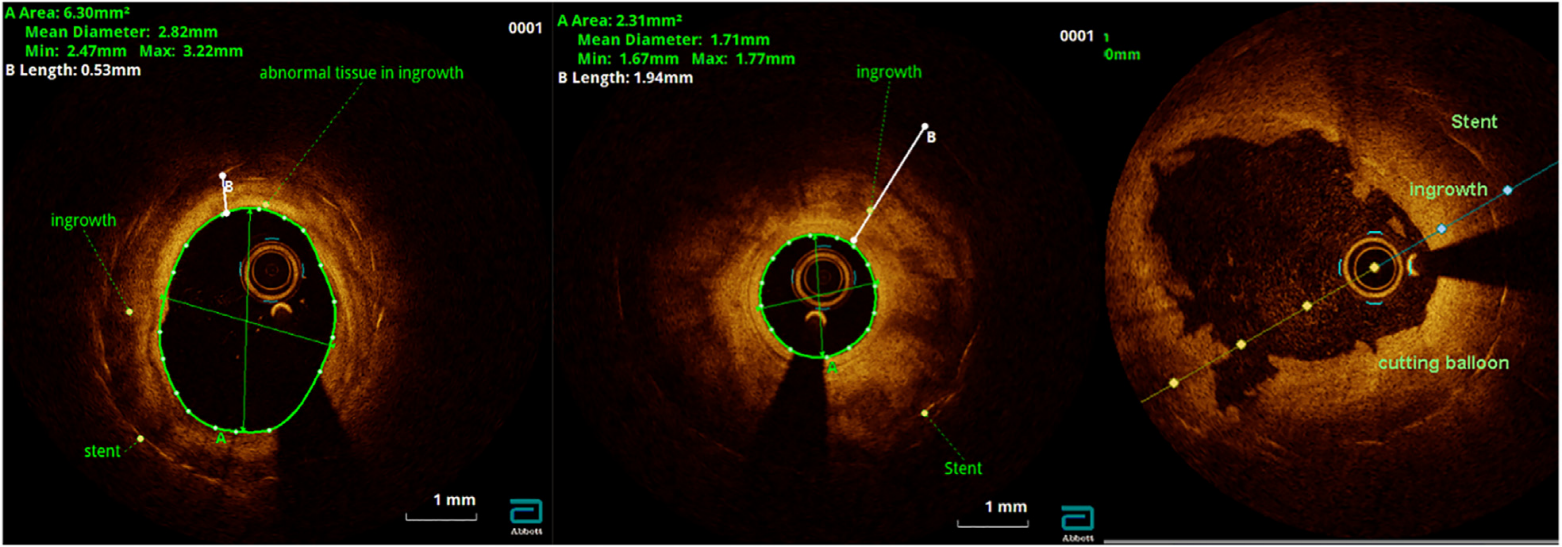
**Optical coherence tomography scan of the pulmonary vein**. Image demonstrates pulmonary vein with a history of stent implantation and current evidence of ingrowth, also known as in-stent restenosis (left, center). There are several areas of wall injury following cutting balloon venoplasty (right).

A feasibility study on the use of optical coherence tomography in infants with advanced PVS was recently reported (Figure 4). It has the advantage of offering high-resolution imaging quality but requires replacing the local blood pool with saline.^39^ Although the clinical application is yet to be determined, Zablah et al^39^ were able to characterize lesions such as intraluminal endothelial tears and dissections that resulted from balloon venoplasty.

### Angiography

**Figure 5:**
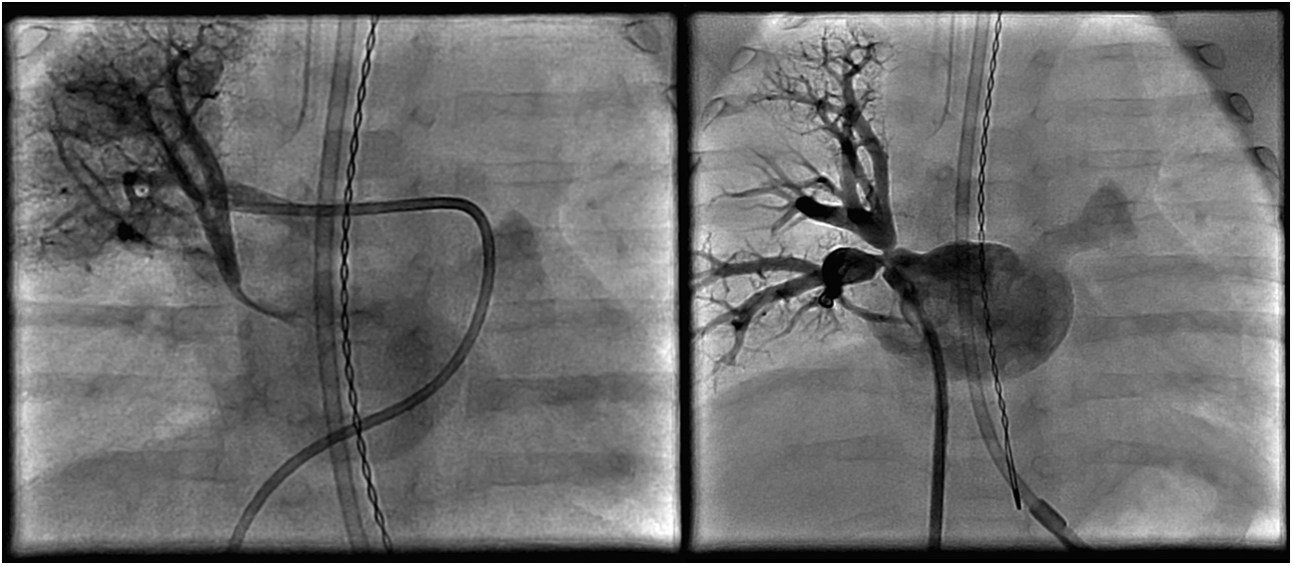
**Comparison of pulmonary artery wedge angiography versus retrograde venography in the right upper pulmonary vein**. Wedge angiography (left) demonstrating possible ostial narrowing of the right upper pulmonary vein. Retrograde venography (right) via long sheath with opacification of the entire right upper and right middle pulmonary veins and better delineation of stenoses distal to the venoatrial junction.

**Figure 6:**
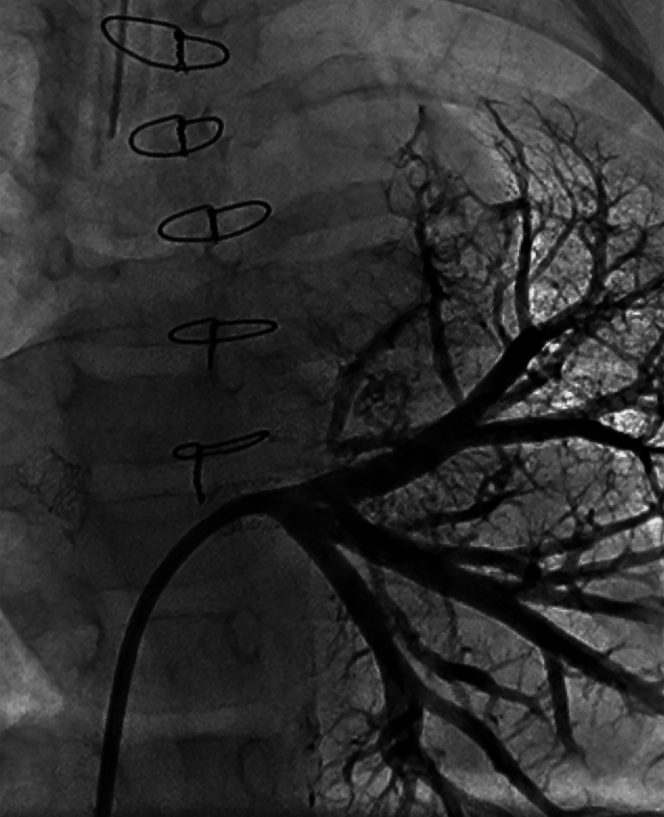
**Left common pulmonary vein venography**. Retrograde venography via guide catheter demonstrates in-stent restenosis, opacification of the entire left lower vein, and atresia of the left upper segment with interlobar collaterals.

Conventional angiography remains the gold standard for evaluating pulmonary venous anatomy and confirming the diagnosis of PVS and is necessary for performing transcatheter interventions. Hemodynamics is first obtained to determine the severity of secondary PH and right-sided heart failure, followed by angiography. Wedge injections into pulmonary artery segmental branches using a balloon-tipped catheter in combination with retrograde injections into the lobar pulmonary veins provide a detailed assessment of the pulmonary venous anatomy and characterize the involvement and extent of disease in each vein. It is noteworthy that retrograde venography offers a distinct advantage over traditional pulmonary artery wedge angiography in its ability to define the entire lobar pulmonary vein anatomy (Figures 5 and 6).

As with CTA, angiographic scoring systems have been developed to characterize the severity of disease in each pulmonary vein.^10^^,^^40^ The baseline vein score and involved vessel location were shown to correlate with vein outcome in a study evaluating the use of imatinib mesylate in a cohort of patients with multivessel PVS.^10^ Further, Patel et al^40^ have developed a thoughtful PVS scoring system to standardize the language for describing PVS.

Transcatheter interventions, specifically venoplasty, can assist in understanding and delineating the mechanism of pulmonary venous obstruction and PVS disease severity. For example, obstructive atrial tissue or vein compression will be compliant without a waist on balloon inflation, a kink will cause the balloon to fold on deflation, and the vessel wall in severe PVS will be noncompliant, milking an inflated balloon into the atrium.

### Surgery

The gross intraoperative appearance of PVS observed at the time of surgical repair demonstrates abnormalities in the affected veins and the surrounding atrial wall. The diseased veins exhibit ostial thickening and luminal narrowing with distal/upstream vasculature ranging from large, thin-walled veins to miniscule strings as a result of venoatrial atresia. The atrial wall surrounding diseased veins has the “off-white” appearance of endocardial fibroelastosis. Other unique features and mechanisms can be delineated, such as slight malposition of the atrial septum with attachments obstructing the right lower pulmonary vein ostium. Samples of pulmonary veins can be sent to pathology for review to confirm the diagnosis.^7^

## Educating the family

It is critical that a thorough counseling session occurs with the family at the time of PVS diagnosis to ensure optimal understanding of the disease and the treatment options. This conversation is akin to the discussion that attends the diagnosis of leukemia in childhood—there is an expectation of survival but with focus and attention paid by the patient’s medical team and medical home. The carefully constructed dialog, which covers the challenging course ahead and the risk of major morbidities and mortality and is balanced by the optimism of the team, requires time, preparation, and humility.^41^ Although the decision to treat PVS initially can appear relatively straightforward, it is necessary to explain that the ramifications of entering the “therapeutic pathway” for PVS require a commitment by both the providers and parents.^42^ Multivessel PVS therapy includes intensive multimodality treatments, including recurrent invasive interventions, polypharmacy, frequent testing/blood draws, radiation exposure, and prolonged hospitalizations (Central Illustration). Although every patient is unique in their clinical course, characterizing the risk profile for each patient on the basis of studied clinical factors can be helpful when educating the family. Patients who are at risk of a poor outcome include those with a younger age and/or lower weight at presentation, a higher number of affected veins, higher severity of disease in the affected veins, severe secondary PH, presence of RV dysfunction, and aspiration.^13^^,^^29^^,^^35^^,^^43^, ^44^, ^45^, ^46^, ^47^, ^48^ Patients with single-ventricle physiology are particularly vulnerable. Patients who respond to therapy thrive, require fewer and less frequent interventions, have shorter hospital stays following reintervention, and have declining RV pressure. PVS disease activity typically “burns out” sometime after 4 years of age, and the long-term survival is determined by the amount of remaining healthy, preserved pulmonary vein bed. Intermittent reinterventions may be required depending on the degree of residual scar within the previously affected veins. Social stressors for families are innumerable and include, but are not limited to, quality-of-life considerations, financial strains (especially if traveling for care), rapid decline in clinical status, lengthy hospitalizations, sibling(s) understanding, and other family life stressors. Expert consultation from palliative care medicine is recommended at the time of diagnosis, and frequent reassessment of patient/parent goals is recommended throughout the treatment course.

**Central Illustration:**
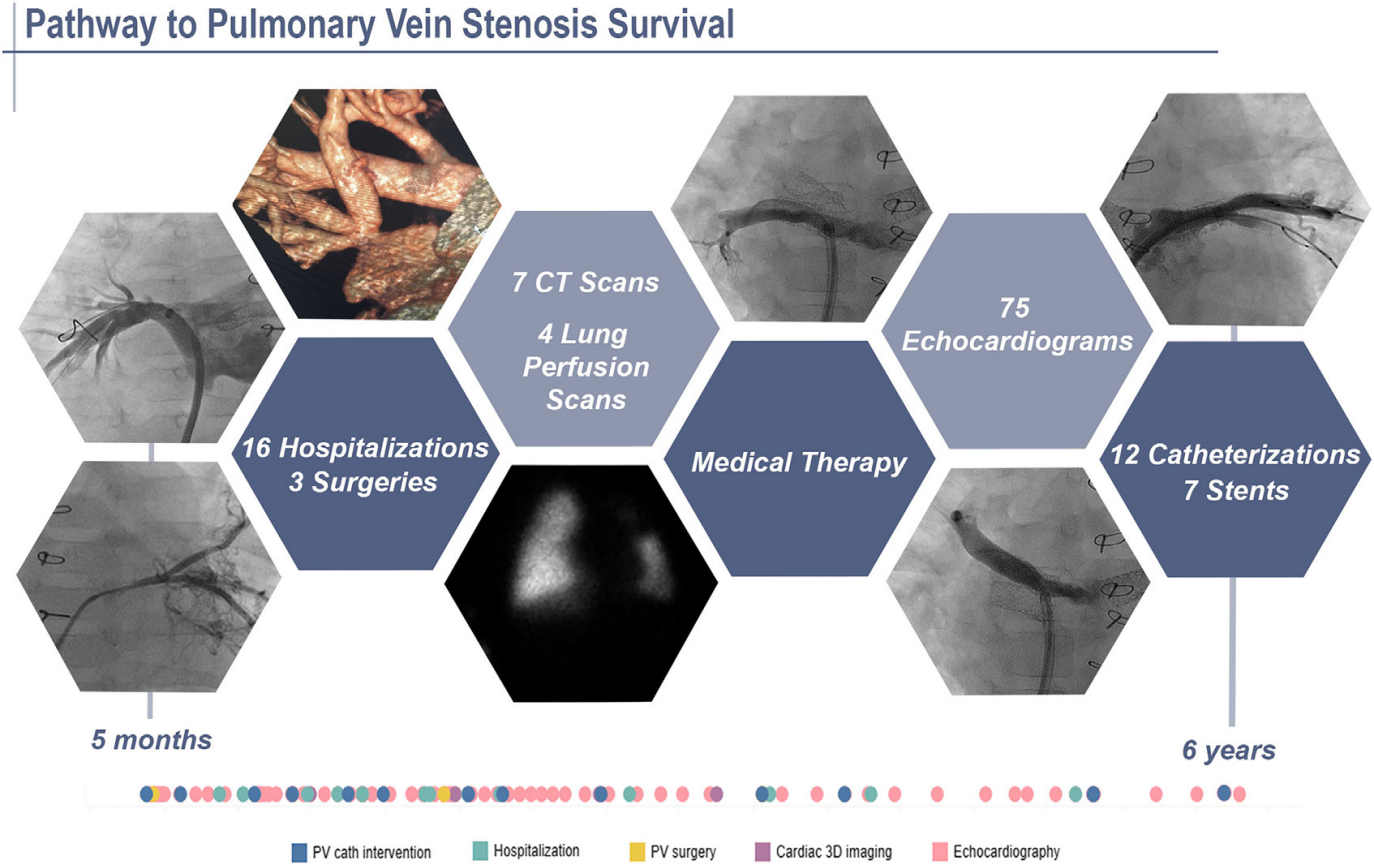
**The pathway to pulmonary vein stenosis (PVS) survival**. The health care interaction timeline demonstrates the clinical path of a 5-month-old infant who initially presented with cardiogenic shock, severe coarctation, left ventricular dysfunction and multivessel PVS. Over the course of 6 years, the patient required numerous transcatheter and surgical pulmonary vein interventions, systemic sirolimus medical therapy, surveillance testing, and frequent hospitalizations. The timeline demonstrates a decreasing frequency of tests and interventions with increasing age, illustrating the impact of therapy on PVS stabilization. 3D, 3-dimensional; cath, catheter; CT, computed tomography; PV, pulmonary vein.

## Treatment

The core principles for the treatment of PVS include the following: (1) diagnose and remove the potential PVS stimulus, (2) identify the anatomic substrate for PVS, (3) establish and maintain normal pulmonary vein pressures by treating PVS lesions using transcatheter and/or surgical techniques, and (4) suppress the proinflammatory state and myofibroblastic activity with medical therapy to prevent or retard PVS recurrence and progression.^10^^,^^31^^,^^49^^,^^50^

### Remove the stimulus

Although the exact etiology of PVS is unresolved, several known diagnoses are associated with either the development of PVS or refractory PVS despite multimodality therapies. Lung disease of any kind, including BPD, interstitial lung disease, malacia, apnea, chronic atelectasis, lower respiratory infections, and aspiration, are associated with PVS.^19^, ^20^, ^21^^,^^31^^,^^48^ Patients with aspiration, in particular, are more likely to have a poor treatment response.^48^ It is unclear whether it is the inflammatory process associated with these conditions that is the initial nidus in the lungs that ultimately drives the fibromyxoid proliferation in the veins. Alternatively, the lung issues may simply be stretching or distorting the pulmonary veins secondary to arterial/bronchial dilation, lung hyperinflation, atelectasis, and airspace disease, which stimulates neointimal hyperplasia. The assumption is that it is a combination of both, and all should be promptly diagnosed and aggressively managed.

The presence of a left-to-right shunt is associated with PVS in premature infants and patients with trisomy 21.^20^^,^^24^ It is hypothesized that excessive overall or lobar Qp (from contralateral pulmonary artery or vein stenosis, for instance) increases WSS in the pulmonary veins, contributing to neointimal hyperplasia.^10^^,^^13^ Using data from catheterization, CTA, and LP scans, Hammer et al^11^ demonstrated elevated WSS in the pulmonary veins of 2 patients with refractory PVS. However, it was not until the WSS normalized that stabilization occurred. Therefore, significant left-to-right shunts are closed at the time of PVS diagnosis in an effort to mitigate PVS progression. “Fenestrated” closure is performed for atrial septal defects to allow access for future transcatheter interventions. Additionally, significant pulmonary artery stenosis, if present, is treated to normalize pulmonary blood flow distribution.

### Identify the anatomic substrate

Identifying the mechanism of obstruction is critical in the management of PVS. Although not all pulmonary venous obstructions lead to neointimal hyperplasia, most pulmonary veins in which PVS develops have an underlying anatomic and/or physiologic substrate that causes disturbed flow. Examples include the following: (1) postoperative PVS with inadequate surgical anastomosis, surgical scar, or residual atrial tissue; (2) normally connected pulmonary veins with distortion from the surrounding anatomy; (3) vulnerable anatomy such as a left common pulmonary vein and a dilated left atrium (ventricular septal defect or mitral regurgitation), which exacerbates the vein compression between the posterior aorta and anterior atrium; (4) unique normally connected pulmonary veins prone to kinking at entry, particularly if cardiomegaly or atelectasis causes heart rotation; (5) stretched pulmonary veins such as those that occur in the left-sided pulmonary veins of patients with scimitar syndrome and dextroposition; or (6) extension of the atrial septum into the ostium of a right-sided pulmonary vein. A combination of cardiac angiography, CTA, and/or IVUS is necessary to fully understand the anatomic substrate for PVS.

### Treat the stenosis

The mainstay of PVS treatment is the ability to effectively relieve the stenosis (and restenosis) using transcatheter and surgical interventional techniques with the goal of minimizing postintervention gradients and encouraging laminar flow. Given the unique geometric shape and surrounding anatomy of individual pulmonary veins (ie, right upper, left upper, etc.) and the heterogeneity of pulmonary venous anatomy among patients, there is no one-size-fits-all intervention for all pulmonary veins. Decision making is further complicated by the lack of randomized studies comparing treatment techniques among similar populations. The “best” type of intervention and reintervention depends on the mechanism of obstruction and the experience of the treating institutions and individual operators. With this in mind, the general principles of surgical and transcatheter interventions for PVS are discussed.

The timing and type of surgical repair of pulmonary venous obstruction are contingent on the mechanism of obstruction, the pulmonary vein anatomy and disease severity at the time of presentation, and the experience of the treating institution. PVS in patients with normally connected veins that have not undergone a previous surgical intervention is commonly offered surgery at presentation. Proximal/downstream lesions with preserved distal/upstream vasculature are ideal compared with more diffuse diseases.^13^ Further, surgery is preferred for geometrically distorted veins with neointimal proliferation not amenable to balloon dilation only.^12^ In patients with pulmonary venous obstruction following anomalous pulmonary venous connection repair, reoperation is performed if the obstruction is localized to the confluence anastomosis or secondary to residual atrial tissue, as seen in the cardiac subtypes (coronary sinus or superior vena cava/right atrium junction). The sutureless technique is the most common technique used for pulmonary venous obstructions, is superior to conventional repairs, and has a lower incidence of restenosis if performed at the time of the neonatal anomalous venous connection repair.^2^^,^^10^^,^^13^^,^^51^^,^^52^ Feins et al^12^ reported a single-center experience with the “anatomic repair,” which extends the left atrium laterally to the pulmonary veins distal to the stenosis, initially with homograft patch plasty but more recently using an autologous atrial flap. The technique demonstrated a survival benefit compared with sutureless techniques but without a decrease in postoperative reinterventions. Although not reported, there is a growing experience of the anatomic repair being performed in combination with complete/partial stent removal for refractory in-stent restenosis.

Most physicians managing PVS believe that transcatheter interventions are the mainstay of therapy for PVS, although there is debate within the field regarding the type and timing of interventions. A minimalistic, less invasive approach can be applied when stimuli for PVS are identified and overcome. For instance, for an ex-premature infant with a history of BPD in which the chest radiography has normalized, is off or nearly off oxygen, and has 2-vessel PVS without severe secondary PH, only balloon venoplasty is a reasonable first-line approach. On the contrary, there are patients with refractory PVS with poor outcomes despite maximum medical and interventional therapies (surgery, balloon, stent, etc.). The middle ground remains gray but general principles can be applied, and institutions should rely on their individual strengths, with the option of receiving outside consultation and/or referral to more experienced centers as needed. In general, transcatheter interventions are indicated for restenosis following surgical repair, rehabilitating distal/upstream disease (including vein atresia), and as primary therapy per institutional preference. Both conventional and cutting balloon venoplasty can relieve vein stenosis with no clear advantage with regard to preventing restenosis.^53^ The goal of venoplasty is to perform an effective dilation, defined as the resolution of the waist with an appropriately sized balloon that provides angiographic resolution of the stenosis. PVS lesions early in the disease course and “burned out” lesions, suspected to be scar, in early school–aged kids tend to resolve with low- to high-pressure venoplasty. Some native and postoperative lesions, especially those that underwent patch plasty and veins status post stent implantation, may require ultrahigh-pressure balloons for effective dilation. Cutting balloon venoplasty may be required for resistance lesions, when diseased veins become noncompliant and milk out conventional balloons and for treating in-stent restenosis. Stent implantation is indicated for a severe residual obstruction/gradient despite effective balloon venoplasty, recurrent PVS despite effective balloon venoplasty, following the recanalization of vein atresia, and large veins of at least 8 mm in diameter.^54^, ^55^, ^56^ Drug-eluting stents are superior to bare metal stents with regard to the in-stent restenosis growth rate and should be considered if a stent is placed in a vein measuring <6 mm.^57^ It is noteworthy that stent implantation requires reintervention for stent redilation until the vein is adult-size, it is not free of refractory in-stent restenosis, and not all drug-eluting stents are guaranteed to fracture as is required to achieve diameters of >8 mm.

### Suppress the disease activity

Targeted medical therapy for PVS aims to prevent, slow, or stop the myofibroblastic activity responsible for the neointimal proliferation and the luminal narrowing in PVS. Patients with at least 2-vessel PVS confirmed with angiography, IVUS, and/or surgical biopsy at the time of pulmonary vein repair are considered candidates for medical therapy. Patients who are at low risk (older at diagnosis, with fewer vessels involved, no significant PH, and normal RV systolic function) whose potential PVS stimulus is identified and overcome can be observed after intervention without medical therapy until restenosis occurs. Imatinib mesylate targets platelet-derived growth factor receptors on the surface of the myofibroblast-like cells and, when given as part of a multimodality approach, is associated with a decreased mortality rate.^7^^,^^8^^,^^12^^,^^58^ Patients who receive at least 90% of their doses were less likely to require reinterventions.^10^^,^^31^ Bevacizumab is another tyrosine kinase receptor inhibitor (antivascular endothelial growth factor receptor) and is considered for patients who are at a high risk (ex-full-term, structurally normal heart) and those with PVS progression while receiving imatinib mesylate.^31^ The risk–benefit ratio of bevacizumab should be considered in all patient populations, including premature infants who rely on vascular endothelial growth factors for organ development.

Systemic sirolimus therapy is currently being used at some centers either to combat in-stent restenosis (Figure 6) or as primary immunomodulatory therapy to retard PVS progression and recurrence.^50^ Sirolimus curbs the proliferation and migration of local smooth muscle cells via mTOR inhibition and decreases neointimal growth rate following transcatheter reintervention.^59^ When used to treat in-stent restenosis, an 8-week course of sirolimus may be prescribed. When used as the primary therapy for PVS, on the basis of recent evidence, sirolimus may be used for 12 to 24 months and should be paired with frequent surveillance and reinterventions.^50^

All medical therapies should be provided under the guidance of an experienced medical provider and/or oncologist. Regular laboratory surveillance is required to monitor for drug toxicities and, in the case of sirolimus, therapeutic levels. The evidence supporting their use in PVS is from nonrandomized cohorts without true control groups. Therefore, caution should be maintained when considering the adoption of these therapies for entire PVS cohorts. Finally, regardless of the manner and method of primary medical therapy, close anatomic surveillance and therapy (transcatheter or surgical) need to be maintained to optimize outcomes.

## Surveillance

Critical to the management of PVS is monitoring for restenosis or new stenosis in previously unaffected veins. The most aggressive disease can develop restenosis in weeks after an intervention. However, most active patients initially recur every approximately 6 to 8 weeks, then approximately 3 months, and then approximately 6 months, until stabilization occurs. Under the assumption that all patients have aggressive disease at the time of diagnosis, monthly clinical assessment and testing are performed for 3 months and then spaced out if no restenosis occurs. This is repeated following any reintervention for restenosis or after discontinuation of medical therapy. The type of noninvasive testing varies by center and includes transthoracic echocardiograms (sedated if necessary) paired with LP scans or serial CTA and, less commonly, serial CMR imaging. Clinical symptoms of restenosis are similar to the initial presentation and include new or worsening respiratory symptoms, feeding intolerance, irritability, and/or fever of unknown origin. Patients presenting with fever are ruled out for infection before reintervention unless they are critically ill and require pulmonary vein reintervention for survival. Echocardiographic evidence of pulmonary vein restenosis includes new or worsening pulmonary vein gradients not confounded by anemia, agitation or obstructive hardware, increasing RV pressure, and/or worsening RV dysfunction. Restenosis by LP scan is represented by at least a 10% decrease in LP if unilateral disease and/or by a subjective decrease in lobar perfusion. Of note, the LP scan may not change if there is simultaneous 4-vessel restenosis. Further, the test can be confounded by pulmonary artery stenosis and by temporary lung issues such as airspace disease or atelectasis.

## Managing symptoms

Diuretics are used for the clinical sequelae of pulmonary edema. Multiple agents with electrolyte replacement therapy may be required in patients with 3 to 4 vessel involvement. Quantities needed for symptom relief are quite dynamic and are based on disease activity and timing of last intervention; nadir typically approximately 2 weeks following the last intervention and escalation, including the possible need for intravenous therapy leading up to an intervention. Patients with severe disease involving the atrial wall and/or patients with a history of extensive PVS repairs may develop left atrium noncompliance, requiring chronic diuretic therapy even after PVS stabilization.

Infants and children with PVS experience poor growth likely due to a combination of high-caloric expenditure, feeding intolerance from right-sided heart failure, reduced gut perfusion, and the side effects of polypharmacy. Patients may require greater calories to achieve anabolic metabolism (eg, >120 kcal/kg/day) and most require gastrostomy tube placement to reach this goal. Close collaboration with a nutritionist as well as a pediatric gastroenterologist may be necessary. Further, a feeding evaluation is performed at diagnosis and following surgical interventions with the goal of eliminating the risk of aspiration.^48^

In severe PVS cases, hemoptysis can occur as a result of alveolar or capillary hemorrhage or airway collaterals from vein atresia and insufficient pulmonary venous egress. Treatment is largely supportive—including positive-pressure ventilation—but may result in the need for a pneumonectomy when vein patency cannot be achieved. However, pneumonectomy is truly a last resort solution reserved for refractory hemoptysis, given the intensity of the surgery, the risk of scoliosis, and the resultant intrathoracic shifting, potentially worsening contralateral PVS.

## Comorbidities

PVS may present in isolation or be associated with several other comorbidities with substantial overlap. Frequently, the presence of one or other of those comorbidities leads to screening during which PVS is identified, at which point PVS may become the predominant diagnosis. Many of those associations have been addressed previously in this review but can be categorized in the following manner:

### Prematurity

Although etiologically unclear at this time, prematurity is closely associated with PVS and should be part of serial screening in this population. In addition, as more centers have adopted protocoled programs for composite evaluation and management in the premature and extremely premature population, echocardiography as the primary screening tool should include a composite evaluation of all pulmonary veins with each incident scan. The totality of the short- and long-term impact of prematurity is not within the scope of this article but includes cognitive delay, cerebral palsy, chronic lung disease (BPD) and associated airway abnormalities, retinopathy of prematurity, hearing impairment, and an increased risk of necrotizing enterocolitis.^60^ Not infrequently, therapies required for those associated conditions may impact the treatment of PVS and need to be considered regarding the timing of testing and cardiac catheterization and any adjuvant therapies that may be indicated. In particular, lower respiratory infections or other septic events could result in the delay of planned or protocoled interventions on pulmonary veins.^60^

### PH

PH frequently complicates PVS in infants with a history of prematurity and chronic lung disease and may exacerbate an underlying substrate that is likely to predispose them to the development of high pulmonary vascular resistance.^61^ In 1 multicenter cohort, two-thirds of patients with PVS presented with PH at a mean age of 6.5 months.^29^ Diagnosis of RV hypertension and PH concomitant with that of PVS presents both a diagnostic and therapeutic dilemma. Although no specific guidelines exist regarding acute vasodilator testing for this population, caution should be employed when performing reactivity testing during cardiac catheterization.^62^ Particular attention should be paid to a potential diagnosis of pulmonary veno-occlusive disease in premature infants with chronic lung disease/BPD with worsening PH despite no obvious single- or multiple-vessel disease. Although acute segmental pulmonary edema may result from vasodilation upstream from an obstructed pulmonary venous segment, rare case reports have described the successful use of inhaled nitric oxide in isolated instances.^63^ The mainstay of therapy for PH in PVS remains the alleviation of the mechanical obstruction. Equal distribution of pulmonary blood flow across a vascular bed with equilibration of resistance provides the ideal scenario for the most effective application of drug therapy. Further, the alleviation of ventilation/perfusion mismatch inherent to PVS optimizes mechanical (or spontaneous) ventilation, normalizing physiology to the extent possible and maximizing response to vasodilator therapy. To date, pulmonary vasodilator drugs have not been approved by the United States Food and Drug Administration for use in World Health Organization–defined group III PH (PH secondary to developmental lung disease). However, the postcapillary nature of PH resulting from PVS has received some attention, and drug therapy may be indicated.^64^ RV hypertension reflects either PVS progression or associated secondary PH—both of which require some form of therapy. Without intervention, RV hypertension can lead to restricted cardiac output and death.^47^ Although historically controversial, there is growing evidence suggesting not only the safety of pulmonary vasodilators in the setting of PVS but also efficacy and utility in patients with severe PVS.^47^^,^^50^^,^^65^ Any use of standard pulmonary vasodilators in this population should be done so with due caution and carefully planned reevaluation to determine the efficacy or progression of PVS. Pulmonary vasodilators are never indicated with diffuse, symmetrical PVS, similar to the contraindication with the pulmonary veno-occlusive disease. PH with deterioration of RV function in the presence of unremitting PVS and maximal tolerated pulmonary vasodilator therapy should provide justification for the evaluation of lung transplantation.^46^

### Genetic syndromes

Genetic analysis of large cohorts of patients with PVS remains nascent. However, a single-center cohort of 71 patients with available material demonstrated identifiable abnormalities in one-third of those individuals. In that cohort, trisomy 21 was the commonest finding (57%), followed by Smith-Lemli-Opitz syndrome (22%).^25^ Comorbidities with known concomitant syndromes would be evaluated in light of those related primarily to PVS.

## Summary

Children with rare, life-threatening disease such as PVS appear to benefit from establishing focused clinical programs that can surveil during the most critical at-risk time points.^66^ Such programs fulfill a number of purposes: (1) creation of evidence-based treatment and surveillance algorithms, (2) delivery of consistent care based on enhanced attention and observance of said algorithms, and (3) careful and intentional introduction of novel therapies in particularly vulnerable members of the cohort. Patients with PVS likewise benefit from a focused, multidisciplinary team with specialists from a broad spectrum to support not only the cardiac, pulmonary, and circulatory sequelae of this disease but also the developmental, gastrointestinal, and pharmacologic complications.

Much remains to be discovered in the field of pediatric PVS. Fortunately, there are now >10 pediatric PVS programs in the United States dedicated to improving PVS outcomes. Emerging efforts include the recent Pulmonary Vein Stenosis Symposium—a 2-day meeting held at Atlanta in 2021—and the creation of multicentered collaborations to collect data and share lessons (www.pvsnetwork.org). Collaborative efforts such as these will be critical in accelerating discoveries and optimizing outcomes in this challenging population.
